# Multifunctional flexible contact lens for eye health monitoring using inorganic magnetic oxide nanosheets

**DOI:** 10.1186/s12951-022-01415-8

**Published:** 2022-04-27

**Authors:** Maowen Xie, Guang Yao, Tianyao Zhang, Qian Wang, Xiaoyi Mo, Qiwei Dong, Wenhao Lou, Fang Lu, Taisong Pan, Min Gao, Dawei Jiang, Kangning Zhao, Yuan Lin

**Affiliations:** 1 School of Materials and Energy, University of Electronic Science and Technology of China, Chengdu, 610054 Sichuan China; 2 State Key Laboratory of Electronic Thin films and Integrated Devices, University of Electronic Science and Technology of China, Chengdu, 610054 Sichuan China; 3 Medico-Engineering Cooperation on Applied Medicine Research Center, University of Electronic Science and Technology of China, Chengdu, 610054 Sichuan China; 4 Department of Nuclear Medicine, Union Hospital, Tongji Medical College, Huazhong University of Science and Technology, Wuhan, 430022 China; 5 State Key Laboratory of Advanced Technology for Materials Synthesis and Processing, International School of Materials Science and Engineering, Wuhan University of Technology, Wuhan, 430070 Hubei China

**Keywords:** Flexible contact lenses, Eyeball movement, Intraocular pressure, Glucose detection, Magnetic oxide nanosheets

## Abstract

As a non-invasive innovative diagnosis platform, advanced flexible contact lenses can dynamically monitor vital ocular indicators, spot abnormalities and provide biofeedback guidance for real-time diagnosis and rehabilitation tracking of chronic eye diseases. However, most of the state-of-the-art reported contact lenses either can only monitor a single indicator at a time or realize multifunctional integration based on multiple materials. Herein, we developed a flexible multifunctional contact lens based on inorganic γ-Fe_2_O_3_@NiO magnetic oxide nanosheets, which can be attached conformally and seamlessly to the eyeball to simultaneously monitor glucose level in tears, eyeball movement, and intraocular pressure. The optimized contact lens has a reliable glucose detection limit (0.43 μmol), superior eye movement measurement accuracy (95.27%) and high intraocular pressure sensitivity (0.17 MHz mmHg^− 1^). This work presents a concept in the biochemical and biophysical integrated sensing of ocular signals using contact lens via an innovative material, and provides a personalized and efficient way for health management.

**Graphical Abstract:**

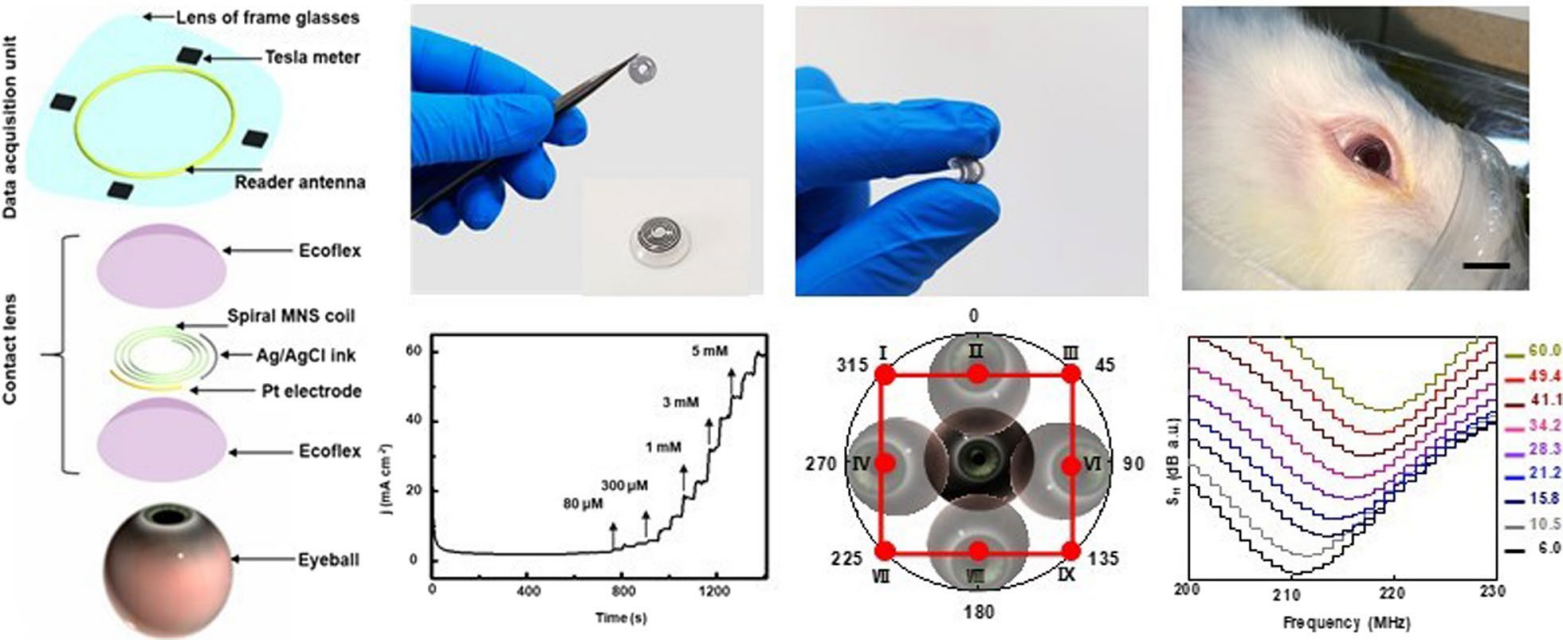

**Supplementary information:**

The online version contains supplementary material available at 10.1186/s12951-022-01415-8.

## Introduction

Chronic eye diseases, including diabetic retinopathy [1–3], glaucoma [4–6], eye divergence [7], etc., are the major causes of visual impairment and blindness. According to the first *World Vision Report* of the World Health Organization, over 2.2 billion people are plagued by visual diseases in 2019 [8]. Timely medical intervention based on real-time diagnosis can prevent the deterioration of these chronic eye diseases. However, abnormal ocular indicators and disease conditions are challenging to monitor and diagnose dynamically only through periodic physical examination [7]. For example, the current clinical measurement of glucose level, intraocular pressure (IOP), and divergence detection still relies on large-sized equipment and empirical diagnosis of medical professionals [9, 10]. Moreover, these approaches can only collect instantaneous data instead of reflecting ocular disease progression and health status, leading to miss the optimal therapy timing and prolong the treatment cycle. Therefore, new monitoring and diagnosis strategies are demanded to render eye health care more operable, predictive, and efficient.

Flexible contact lenses, as a non-invasive wearable accessory, provide a promising development platform for eye health monitoring and disease diagnosis [11–16]. Recently, biochemical or biophysical sensors have been integrated into contact lenses to detect biomarkers in tear fluids [11, 13, 17], eyeball movement [7], mechanical deformations [9], etc. Among various biochemical markers, glucose is essential for diabetes diagnosis. Glucose oxidase-based electrochemical method is the primary detection means [18–20], while organic reagents are easily contaminated, inactivated or degraded, making it difficult to exert stable performance in dynamic environments [21]. In the recent boom of nanomaterials and biomedical engineering, non-enzyme sensors have brought innovative developments to current glucose sensors. Non-enzyme sensors based on inorganic or hybrid materials have achieved good sensitivity and showed great potential for clinical applications [22]. However, to realize biochemical and biophysical hybrid sensing functions, multiple materials-based complex units must be integrated into the limited area, which inevitably increases the contact lenses’ hardness. Thus, innovations in materials and device designs are essential to developing multifunctional flexible smart contact lenses with high accuracy, precision, and reproducibility. Magnetic nanomaterials (such as Fe, Cr, Gd, Co, Mn, Ni, and their chemical compounds) can respond to an external magnetic field due to their special superparamagnetic property. Among them, γ-Fe_2_O_3_ and NiO are expected to be ideal candidates for long-term clinical applications due to their stable magnetic properties and good electrocatalytic activity for glucose oxidation, respectively [22, 23].

In this work, a multifunctional contact lens (MCL) was developed employing inorganic γ-Fe_2_O_3_@NiO magnetic oxide nanosheets (MNS) as the sensing material, which can simultaneously monitor biochemical glucose level in tears, biophysical eyeball movement and IOP. We synthesized γ-Fe_2_O_3_@NiO nanosheets on nickel foam *via* a one-step hydrothermal reaction and successfully fabricated MCL using flexible electronic technologies. The nickel foam with a three-dimensional web porous structure provides high conductivity and specific surface area for MNS, which provides the possibility of superb glucose catalytic activity. The as-prepared MNS-based enzyme-free glucose sensor exhibited a low detection limit (0.43 µmol) and wide linear range (0.005–6.0 mM) in the traditional electrochemical three-electrode detection. The magnetic field distribution of the MNS unit has a positional difference when the eyeball moves, and the magnetic intensity of the external tesla meter can reproducibly reflect the real-time eye vergence signal. Furthermore, the designed spiral MNS functional unit deforms with the fluctuation of IOP, which sensitively and linearly affected the characteristic frequency point of the external reader antenna. In general, the flexible MCL based on MNS can simultaneously detect biochemical and biophysical signals, which has high-efficiency application prospects for eye health monitoring and disease diagnosis.

## Results and discussion

### Design and working principle of the MCL

**Fig. 1:**
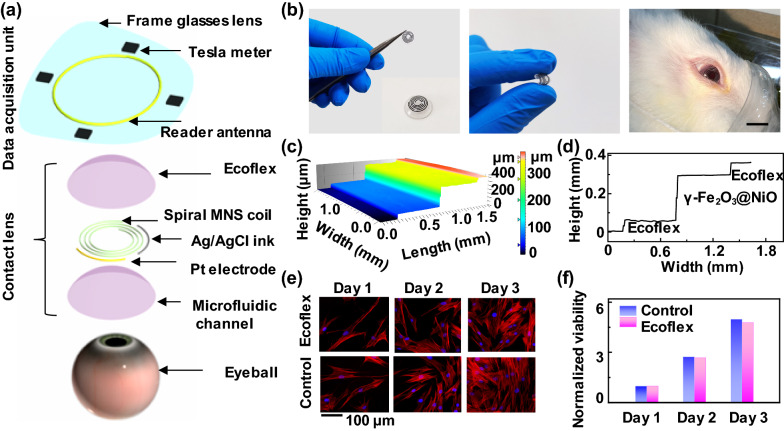
Design and working principle of the MCL. **a** Schematic diagram of key components of the MCL and the data acquisition unit. **b** Optical images of the MCL in the initial state (left) and the bending state (middle), and the experimental setup on the rabbit eye (right). Scale bar, 1 cm. **c** Three-dimensional thickness diagrams of the contact lens and **d** Corresponding thickness ladder diagram. **e** Fluorescence images of stained cells cultured on a regular cell culture dish and the surface of Ecoflex package. **f** Comparison of normalized cell viability for three days showing excellent biocompatibility of the packaged device (n = 3 for each group). All data in **f** are presented as mean ± s.d

The eye health monitoring system consisted of two modules: a MCL for physiological eye signals sensing and a data acquisition unit integrated on a frame glasses lens. The structure details and key components of the MCL and data acquisition unit are shown in Fig. 1a. A 5-turn spiral configuration was designed for MNS to ensure flexibility while not blocking eye vision. For glucose detection in tears, MNS was served as the working electrode in the three-electrode electrochemical test system. Platinum (Pt) wire and silver/silver chloride (Ag/AgCl) ink electrode were integrated into the same plane to act as the counter electrode and the reference electrode, respectively. The biocompatible Ecoflex was used as top and bottom encapsulation layers, and the bottom encapsulation layer has microfluidic channels to collect tears in one direction [24]. The detailed fabrication process and configuration parameters of the MCL are illustrated in Additional file 1: Fig. S1. Four probes of tesla meters and a reader coil of the vector network analyzer were integrated on the frame glasses lens to build the data acquisition unit. Four-tesla meter probes were distributed at the midpoints of the four sides of the external frame glasses lens to detect the changes in magnetic intensity with the eyeball movement. The circular copper reader coil can monitor the deformation of the spiral MNS along with the fluctuation of IOP based on mutual electromagnetic coupling. The overall dimensions of the initial-state curved MCL are approximately Φ12 mm × 0.35 mm (Fig. 1b, left), which can be bent to 180 degrees without structural damage (Fig. 1b, middle). The mechanical robustness of the flexible MCL was tested by a pressing system using a spherical plastic ball with a radius of 4 mm (Additional file 1: Fig. S2a–c). The finite element analysis (FEA) results demonstrate that the strain (< 0.45%) was consistently smaller than the failure strain (~ 3%) even when the MCL was pressed up to 10 mm. Both the FEA results and corresponding height experimental results exhibited a similar deformation trend (Additional file 1: Fig. S2d, e). Meanwhile, the MCL was repeatedly pressed 200 times without causing any damage, which confirmed the good mechanical robustness of the MCL (Additional file 1: Fig. S2f). In addition, the average vertical and horizontal bending stresses of the MCL and BCL were measured by three-point bending to reveal their mechanical property. No significant difference in bending stress was observed between the MCL and BCL (Additional file 1: Fig. S3). These results reveal the good mechanical properties of MCL [25]. The flexible and mechanical properties ensure that the MCL can be conformally and seamlessly attached to the rabbit eyeball (Fig. 1b, right). The top- and side-view three-dimensional microscope scanned images were shown in Fig. 1c, d and Additional file 1: Fig. S4, respectively. A cross-sectional height profile was taken along one scanning line to quantify the multi-layered geometry. The average thickness of the top Ecoflex encapsulation layer, MNS, and bottom Ecoflex encapsulation layer are 55 μm, 235 μm, and 55 μm, respectively. Then, the fibroblasts were cultured on the surface of the encapsulated MCL and in a petri dish for three days to confirm the biocompatibility. Fluorescence staining results (Fig. 1e, f**)** showed that the cell density and morphology of the two culture media were similar, and there was no cell death or distortion on the surface. To further evaluate the biosafety of the material, the encapsulated MCLs were worn on the left eyes of the rabbits for 8 days. In general, with increasing wearing time, all the rabbit corneas did not show any abnormality, suggesting that the MCL are highly biocompatible and safe. Furthermore, after wearing MCL for 0 days, 4 days and 8 days, the rabbit corneas were collected for histological examination by hematoxylin–eosin (H&E) staining. The results showed no signs of pathological inflammations for at least 8 days of wearing, indicating the non-toxicity and biocompatibility of the wearable MCL (Additional file 1: Fig. S5).

### Inorganic MNS characterization

**Fig. 2:**
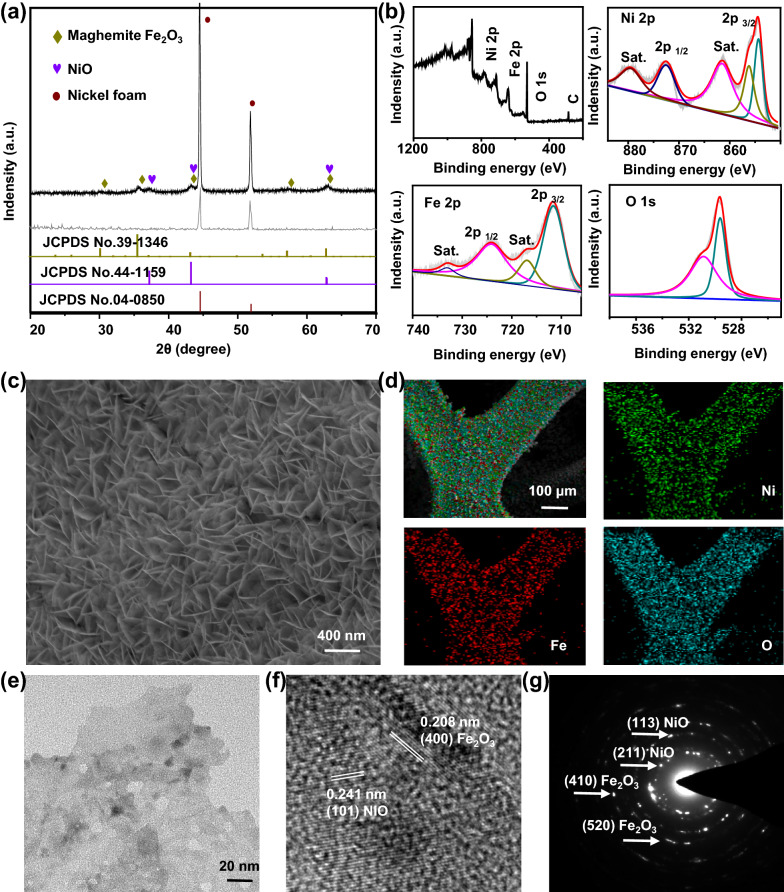
Characterization of the as-prepared MNS.** a** The XRD pattern of the as-prepared MNS compared with the standard data of maghemite Fe_2_O_3_, NiO, and nickel. **b** XPS survey spectrum and XPS spectra in Ni 2p, Fe 2p, and O 1s regions for the as-prepared MNS. **c** SEM image and (**d**) corresponding EDX elemental mapping images for the as-prepared MNS. **e** The TEM image, **f** the HRTEM image and **g** the SAED pattern of the as-prepared MNS

As the core component of MCL, the inorganic γ-Fe_2_O_3_@NiO MNS was first synthesized and characterized. The phases of the as-prepared MNS was detected by X-ray diffraction (XRD). The 2-theta scanning patterns of the as-prepared MNS and the nickel form are shown in Fig. 2a. The detected peaks at 30.2°, 35.6°, 43.3°, 57.3° and 62.9° correspond to the diffraction from the (220), (311), (400), (511) and (440) planes of the γ-Fe_2_O_3_ phase (JCPDS No. 39-1346) [26], while the other detected peaks correspond to the diffraction from planes of the NiO phase (JCPDS No. 44-1159) [27] except for the peaks of nickel foam[28] at 44.5° and 51.8° (JCPDS No. 04-0850). XPS analyses were then utilized to probe the surface chemistry of MNS (Fig. 2b). In the Ni 2p region, the binding energies at 854.0 and 872.1 eV can be attributed to Ni 2p_3/2_ and Ni 2p_1/2_, implying the presence of the Ni^2+^ oxidation states.[29] The peak at 856.1 eV could be indexed to Ni 2p_3/2_ in oxidized Ni species arising from superficial oxidation of the nickel foam[30]. In the Fe 2p region, the peaks at 724.0 and 711.8 eV belong to Fe 2p_1/2_ and Fe 2p_3/2_ of Fe_2_O_3_ [31, 32]. The O 1s spectrum exhibits only a single peak at 529.6 eV, which appertains to the metal-oxygen bonds, and the satellite peak at 531.2 eV comes from oxygen-containing species adsorbed on the surface [33]. All these observations suggest that MNS was successfully prepared. The morphology of MNS was investigated by scanning electron microscope (SEM). It can be seen that the successful formation of MNS on Ni foam (Fig. 2c; Additional file 1: Fig. S6). As revealed by energy-dispersive X-ray spectroscopy (EDX) analysis (Additional file 1: Fig. S7) and corresponding EDX elemental mapping (Fig. 2d), the elements Ni, Fe, and O were identified and evenly distributed in the resulting product. Transmission electron microscopy (TEM) and high-resolution TEM (HRTEM) were employed to further characterize the microstructure of the MNS (Fig. 2e, f). The ultrathin nanosheets have clear lattice fringe with interplane spacing of 0.208 and 0.241 nm corresponding to the (400) plane of γ-Fe_2_O_3_ and (101) plane of NiO, respectively [34–36]. The selective area electron diffraction (SAED) pattern (Fig. 2g) shows several rings made up of discrete spots, which can be indexed to the (113), (211), (410), and (520) planes of MNS structure. In summary, all these characterization results indicated the porous and uniform γ-Fe_2_O_3_@NiO nanosheets were successfully synthesized.

### Biochemical glucose detection

**Fig. 3:**
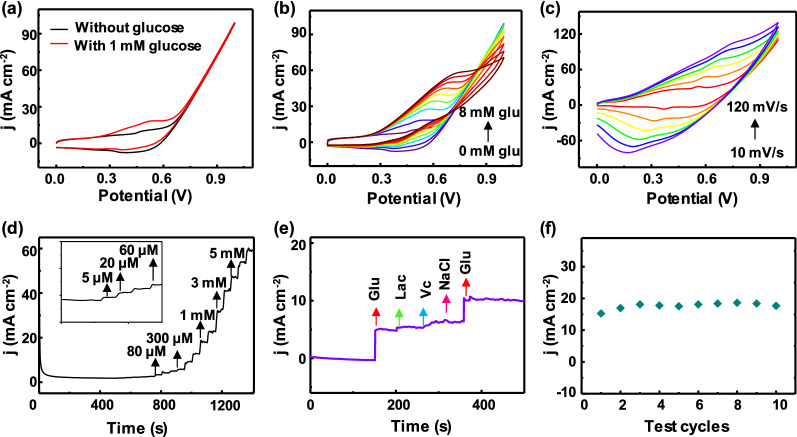
Biochemical glucose detection of the MCL.** a** The CV curves of the as-prepared MNS with and without 1 mM glucose at a scan rate of 10 mV s^− 1^. **b** Current change of the as-prepared MNS electrode in diverse glucose concentrations from 0 to 8 mM. **c** CVs of the as-prepared MNS at different scan rates from 10 to 120 mV s^− 1^. **d** Real-time amperometric responses of glucose concentrations (continuous addition of glucose at an interval of 50 s). **e** Selectivity test of the as-prepared MNS for Lac (1.0 mM), Vc (0.5 mM), NaCl (2.0 mM), and Glu (1.0 mM) at the potential of 0.5 V. **f** The reproducibility of the glucose sensor in different areas of ten samples with 1.0 mM glucose (n = 3 for each group)

The as-prepared MNS on the conductive nickel foam was employed in the enzyme-free glucose sensor. The principle of enzyme-free glucose detection is based on the redox reaction between the metal ions center and the glucose.[37] Fig. 3a shows the cyclic voltammetry (CV) response of MNS in the absence and presence of 1 mM glucose. It can be observed that the current response increases significantly accompanied by addition of glucose, indicating that MNS has a perceptible effect on glucose oxidation. Figure 3b reveals the CVs of MNS in different glucose concentrations, suggesting higher anodic current density with more glucose. Furthermore, the current was varied with different scan rates to indicate the surface reaction of the electrode. As is shown in Fig. 3c, the redox peak current densities of MNS gradually increase with the rising scan rates. A good linear relationship between the peak currents of the anode (R^2^ = 0.99) and cathode (R^2^ = 0.98) proves the diffusion control process of glucose oxidation on MNS (Additional file 1: Fig. S8) [38], where R is defined as correlation coefficient. Figure 3d shows the representative amperometric responses of the glucose sensor by continuously adding glucose at an oxidation voltage of + 0.5 V, and the corresponding current vs. analyte concentration curves are presented in Additional file 1: Fig. S9. The results prove that the amperometric signal has a good linear correlation to glucose concentration in the range of 0.005–0.5 mM (R^2^ = 0.991) and 1.0–6.0 mM (R^2^ = 0.995). The limit of detection (LOD) of the glucose was calculated from the equation: LOD = RSD/slope [39, 40] (RSD is relative standard deviation; the slope is 14.1). As observed, the LOD value of MNS is 0.43 μm. These values compare favorably to the behaviors of most reported electrochemical non-enzyme glucose sensors (Additional file 1: Table S1).

Selectivity is another major factor in assessing the performance of electrodes for non-enzymatic glucose detection [41, 42]. The influence of common interfering species such as lactic acid (Lac), ascorbic acid (Vc), and NaCl were investigated. As is shown in Fig. 3e, the negligible current response of interfering species was observed. In sharp contrast, the current increases rapidly after adding 1.0 mM glucose. Ten synthesized MNS samples were employed to assess the reproducibility (Fig. 3f), the CV curves were performed separately to compare the peak current of each sample under 1 mM glucose. The RSD of 10 measurements was 5%, indicating good reproducibility of this sensor. The above results indicated that the MCL has high sensitivity, selectivity, and reproducibility as a blood glucose monitoring platform.

### Biophysical eye vergence monitoring

**Fig. 4:**
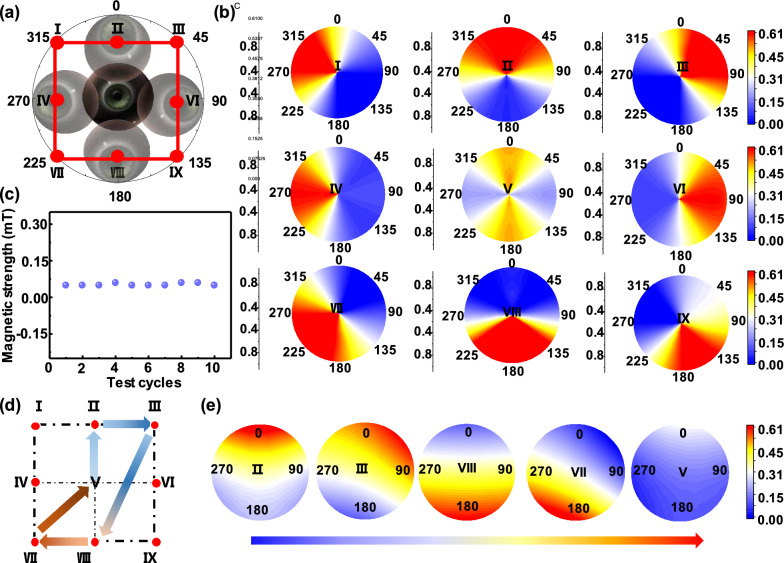
Magnetic intensity analysis of the MCL. **a** Simulated image of eye movement in 9 directions (Direction V is the center point, and each direction is represented by a red dot). **b** The intersection function simulates the magnetic heat map of eye movement in different directions. **c** Repeatability test of magnetic induction intensity (n = 10) **d** Dynamic eyeball movement trajectory and **e** corresponding magnetic heat maps (starting from the center point V)

Eye vergence insufficiency is a common binocular motor disorder. Current treatments are limited by high cost and cannot constantly record a precise eye vergence response [43–46]. Based on the difference of magnetic induction intensity of ferromagnetic materials at different positions of the MCL, we fixed Tesla meters at the four sides of the external frame glasses lens to simulate the movement of magnetic contact lens in nine directions (I–IX), corresponding to eye movements of 0°, 45°, 90°, 135°, 180°, 225°, 270°, 310°, and the center position, respectively (Fig. 4a). Then the intersection function was used to determine and analyze the magnetic intensity heat maps in Fig. 4b (details were shown in Materials and Methods) to achieve the rapid magnetic response of the eyeball movement. To certify the signal quality of eye vergence, we performed ten consecutive magnetic tests on the same material (Additional file 2: Movie S1), which results in an accuracy of 95.27% and a fast magnetic response (Fig. 4c). Finally, the real-time movement of the eyeball in different directions were simulated by continuously moving the MCL and the numerical changes of the external Tesla meter was recorded. Figure 4d shows the trajectory of MCL extracted from the Additional file 3: Movie S2. The magnetic intensity heat maps (Fig. 4e) obtained by the intersection function analysis show that the experimental datas were consistent with the actual movement trajectory, which proved the reliability of the intersection function model to monitor eye movements. The MCL offers highly sensitive detection of eye vergence via intersection function analysis. This MCL enables accurate detection of eye movement for patients with eye vergence, compared with the reported wearable devices, it has the advantages of compactness, portability and low price (Additional file 1: Table S2), which is promising to be further investigated for a clinical study.

### Biophysical IOP monitoring

**Fig. 5:**
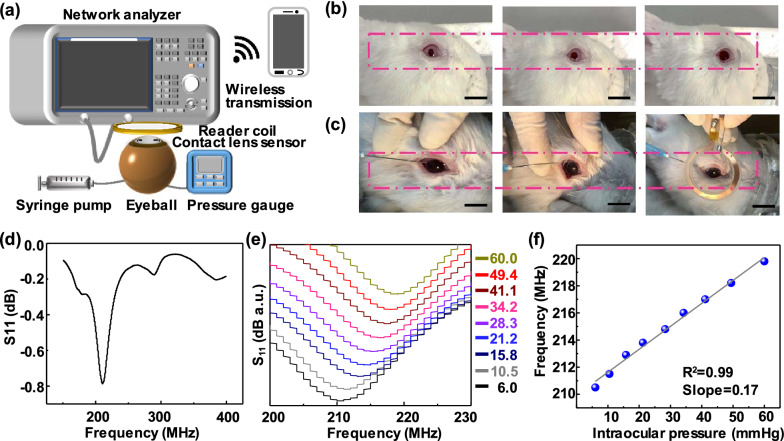
IOP analysis of the MCL.** a** Schematic of the experimental set-up for IOP sensing. A syringe pump controlled the pressure in the eye. The reading coil is placed parallel on the top of the MCL. **b** Photographs of the mydriasis process in the rabbit eyeball. Scale bar, 1 cm. **c** Photographs of the IOP test process on rabbit eye. The injection of physiological saline to control intraocular pressure (left), the wearing of contact lens (middle), and wireless monitoring (right). **d** Reflection coefficients at an intraocular pressure of 6.0 mm Hg after wearing MCL on an eye of a rabbit in vivo. **e** Wireless recording of the reflection coefficients under different pressures (6.0–60.0 mmHg) in the frequency range of 200 to 230 MHz. **f** The frequency response of MCL worn on the rabbit’s eyes at a pressure between 6 mm Hg and 60 mm Hg

Elevated IOP is the most significant risk factor for glaucoma. Thus, real-time tracking of IOP is considered as a promising way to pre-diagnose glaucoma disease (Additional file 1: Table S3) [47, 48]. A resonant inductive wireless energy transfer system for continuously non-invasive IOP monitoring was designed and systematically tested. The principle of the wireless IOP sensor is to use the coupling and mutual inductance between the MCL and the inductance coil [49]. The detailed experimental setup of the IOP sensor is shown in Fig. 5a. The intraocular pressure was changed by controlling the syringe pump and the reading coil was placed in parallel above the MCL. The network analyzer obtains the drift of the resonance frequency. Additional file 1: Fig. S10 shows the IOP test system composed of a portable network analyzer and a smartphone to achieve wireless intraocular pressure sensing. The MCL was tested on a rabbit eyeball, and the rabbit’s eyeball was dilated for 30 min to protect the iris (Fig. 5b). Next, a syringe pump with a needle inserted into the eyeball. After wearing the MCL, physiological saline was injected into the eyeball through the syringe pump to control the intraocular pressure in the range of 6–60 mmHg. Simultaneously, a circular induction coil with a diameter of 40 mm is connected to the network analyzer, and the resonance frequency of the sensor was read wirelessly (Fig. 5c). Figure 5d shows the reflection spectrum collected at 6 mmHg pressure. The apparent reflection peak observed at 210.5 MHz is attributed to the sharp change in the coupling capacitance and inductance. When MCL deforms with the fluctuation of high IOP, the inductance of the coupling capacitor increases, and the reflection intensity value decreases. Figure 5e demonstrates the reflectance spectrum of MCL worn by the rabbit eyeball at 6–60 mmHg, confirming that high intraocular pressure shifts the reflection spectra of MCL to a lower frequency [11]. In addition, the MCL resonance frequency is almost positively linearly correlated with IOP (Fig. 5f) by the slope of 0.17 mmHg in the physiologically relevant range of intraocular pressure. Electromagnetic simulation and experimental results were used to evaluate the effect of eye movements on IOP sensitivity, indicating that the relative deviation of eye movement in 9 directions were much lower than each 1 mmHg increase in intraocular pressure (Additional file 1: Fig. S11). In short, MCL can effectively detect intraocular pressure and have broad application prospects in wireless, dynamic ophthalmological diagnosis.

## Conclusions

In this work, inorganic γ-Fe_2_O_3_@NiO MNS has been successfully synthesized via a one-step hydrothermal reaction and has been used as the sensing material to fabricate contact lens with multifunction integrated. The MCL has excellent performance on glucose detection, eyeball movement, and IOP. As an enzyme-free glucose sensor, MCL exhibits good electrochemical properties with a low detection limit of 0.43 μm and a wide linear range of 0.005–0.5 mM and 1.0–6.0 mM. Meanwhile, the MCL sensor has high accuracy (RSD: 95.27%) and fast magnetic response in eye movement. The feasibility of locating eyeball movement by magnetic strength is proved through theory and experiments, providing more insights for magnetic materials in the field of flexible wearable ophthalmic treatments. Simultaneously, the MCL adopts a spiral structure to test the IOP on a rabbit eyeball. The linear relationship between the resonance frequency and intraocular pressure is obtained by wireless sensing technology. Compared with advanced reported IOP sensors, this work’s sensitivity (0.17 MHz mmHg^− 1^) and detection range (6.0–60.0 mmHg) have comparable performance. Therefore, the MCL can effectively avoid the malignant development of ocular diseases and further preventing the risk of blindness. This work will open up exciting new avenues for exploring magnetic oxides as a means of ophthalmic disease prevention and diagnosis.

## Materials and methods

### Preparation of MNS

0.1 mol Fe(NO_3_)_3_·9H_2_O (Aladdin Ltd. in Shanghai), 0.1 mol Ni(NO_3_)_3_·9H_2_O (Aladdin Ltd. in Shanghai) and 1.0 mmol CH_4_N_2_O (Chengdu Kelon Chemical Reagent Factory) were added into 30 mL deionized water, then transferred into a 50 ml autoclave after stirring uniformly. A fresh nickel foam (2 × 3 cm) was infused into the homogeneous solution. The autoclave was kept at the temperature of 120 °C for 6 h in one electric oven and then naturally cooled to ambient temperature. The material was removed, rinsed several times with deionized water, and dried in a vacuum oven at 60 °C.

### Preparation of spiral MCL

The detailed preparation process is shown in Additional file 1: Fig. S1. First, the prepared MNS was transferred to a thermal release tape, and the polyimide tape was sticked on the glass slide (Additional file 1: Fig. S1a). Then, MNS spiral pattern (line width: 300 μm, inner diameter: 2.4 mm, outer diameter: 8.4 mm) and the microfluidic channel template (line width: 300 μm) on a glass slide were obtained by the laser cutting technique. The Ecoflex solution was spin-coated on the glass slide at a spin-coating speed of 1200 r/min, and then dried in an oven at 80 °C for 30 min to obtain an Ecoflex encapsulation layer containing microfluidic channel (Additional file 1: Fig. S1b). Next, the spiral MNS, Ag/AgCl ink and platinum wire are filled in the bottom flexible Ecoflex with microfluidic channel (Figure S1c). Finally, all of them were transferred into the contact lens mold containing 0.1ml Ecoflex (Additional file 1: Fig. S1d). The functional contact lens was obtained after heating at a constant temperature of 80 °C for 20 min. The thickness of the overall MCL device is about 350 μm.

### Characterizations of the MNS

XRD data were acquired from X’ Pert Pro (PANalytical, Holland) X-ray diffractometer with Cu Kα radiation (45 kV, 30 mA) with the wavelength of 0.154 nm. SEM measurements were performed on ZEISS Gemini 300 scanning electron microscope at an accelerating voltage of 30 kV. XPS spectra were acquired on a Thermo Scientific K-Alpha X-ray photoelectron spectrometer using Al Kα radiation (0.6 eV). TEM spectra were acquired from FEI Tecnai G2 F20. The patterning process of MNS by a laser micromachining system (DelphiLaser Inducer-6001-N). The three-dimension images were photoed by confocal laser scanning microscopy (Zeiss LSM 800).

### Electrochemical measurement

Electrochemical measurements were performed with a CHI 660E electrochemical analyzer (CH Instruments, Inc., Shanghai). All electrochemical tests adopt the traditional three-electrode system, and the experimental results were obtained under normal temperature and pressure.

### Magnetic induction measurement

The mechanism of recording eye movement are as follows:

Assume that the four Teslas are numbered as A, B, C and D in clockwise order. test points 1–9 are sorted in Fig. 4a:

$${\text{F}}\left( {{\text{A}} > {\text{C}}} \right) = \left\{ {{\text{x}}|{{1}},{{2}},{{3}}} \right\} \,\, {\text{G}}({\text{A}} \approx {\text{C}}) = \left\{ {{\text{x}}|{{4}},{{5}},{{6}}} \right\}\, \,{\text{H}}\left( {{\text{A}} < {\text{C}}} \right) = \left\{ {{\text{x}}|{{7}},{{8}},{{9}}} \right\}.$$

$${\text{I}}\left( {{\text{B}} > {\text{D}}} \right) = \left\{ {{\text{x}}|{\text{3}},{\text{6}},{\text{9}}} \right\}\,\, {\text{J}}({\text{B}} \approx {\text{D}}){\text{ }} = \left\{ {{\text{x}}|{\text{2}},{\text{5}},{\text{8}}} \right\}\,\, {\text{K}}\left( {{\text{B}} < {\text{D}}} \right) = \left\{ {{\text{x}}|{\text{1}},{\text{4}},{\text{7}}} \right\}$$

For example, point 1 = F∩K. Similarly, point 2–9 are obtained by analogy.

### Rabbit eyeball experiments

The IOP test was performed using the rabbit eyeball. The needle was inserted into the rabbit eyeball and controlled by a syringe pump. The intraocular pressure of the eyeball was measured by the pressure sensor inserted into the eye chamber. The change of resonance frequency was characterized wirelessly using the network analyzer (Rohde&Schwarz, znb 20).

## Supplementary information

**Additional file1. **Supplementary information for Multifunctional flexible contact lens for eye health monitoring using inorganic magnetic oxide nanosheets.

**Additional file 2. Video S1: **Ten consecutive magnetic response reproducibility tests. 

**Additional file 3. Video S2: **MCL simulating eye movement trajectory.

## Data Availability

All data generated or analyzed during this study are included in this manuscript and its Supplementary Information.
