# Supplementary material for: Multifunctional flexible contact lens for eye health monitoring using inorganic magnetic oxide nanosheets
Source: J Nanobiotechnology. 2022 Apr 27;20:202. doi: 10.1186/s12951-022-01415-8 (PMC9044588; doi:10.1186/s12951-022-01415-8)
Supplement: Supplementary file 1 — Additional file1. Supplementary information for Multifunctional flexible contact lens for eye health monitoring using inorganic magnetic oxide nanosheets. [file 12951_2022_1415_MOESM1_ESM.pdf]

**Supplementary information for**

# Multifunctional flexible contact lens for eye health monitoring using inorganic magnetic oxide nanosheets

Maowen Xie[1], Guang Yao[1,2,3*], Tianyao Zhang[1], Qian Wang[1], Xiaoyi Mo[1], Qiwei Dong[3], Wenhao Lou[1], Fang Lu[3], Taisong Pan[1,2], Min Gao[1,2], Dawei Jiang[4], Kangning Zhao[5], Yuan Lin[1,2,3*]

[1]School of Materials and Energy, University of Electronic Science and Technology of China, Chengdu 610054, Sichuan, China.

[2]State Key Laboratory of Electronic Thin films and Integrated Devices, University of Electronic Science and Technology of China, Chengdu 610054, Sichuan, China.

[3]Medico-Engineering Cooperation on Applied Medicine Research Center, University of Electronic Science and Technology of China, Chengdu 610054, Sichuan, China.

[4]Department of Nuclear Medicine, Union Hospital, Tongji Medical College, Huazhong University of Science and Technology, Wuhan, 430022, China

[5]State Key Laboratory of Advanced Technology for Materials Synthesis and Processing, International School of Materials Science and Engineering, Wuhan University of Technology, Wuhan 430070, Hubei, China.

* Correspondence should be addressed to G.Y. (gyao@uestc.edu.cn) or Y.L. (linyuan@uestc.edu.cn)

## Supplementary Figures

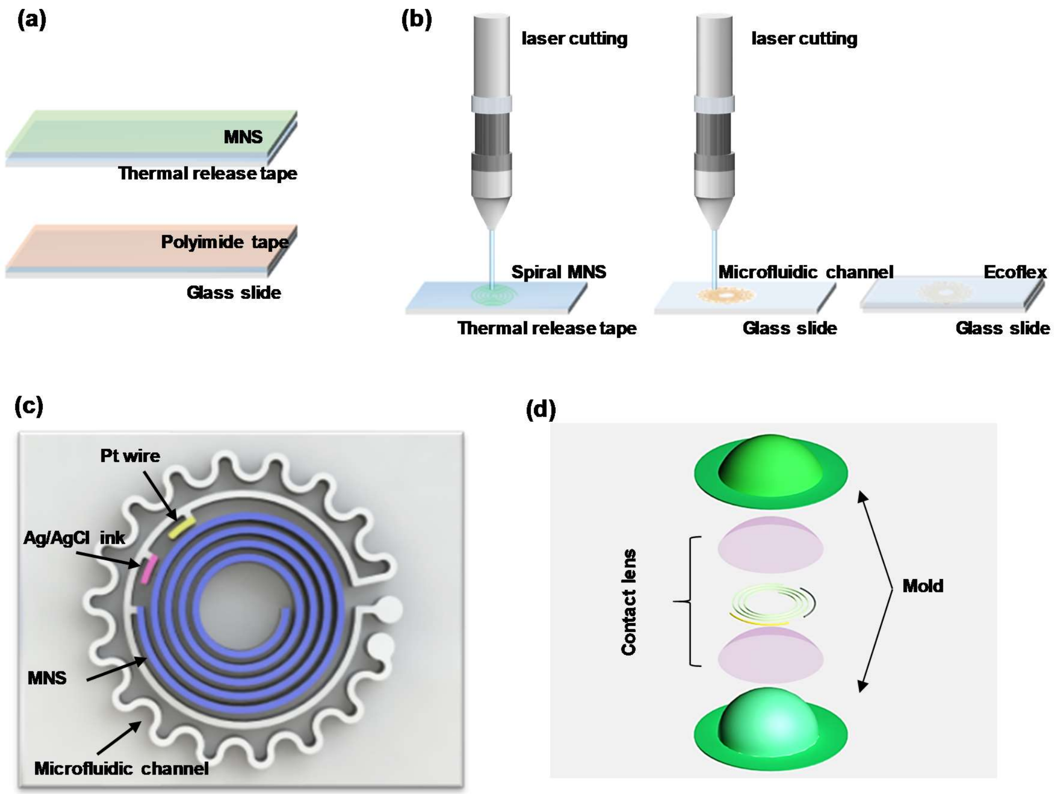


**Figure S1. Preparation of spiral MCL. (a)** Transfer the MNS to the thermal release tape and stick the polyimide tape on the glass slide. **(b)** Laser cut the spiral MNS and the microfluidic channel on the Ecoflex encapsulation layer. **(c)** Transfer electrode materials to the Ecoflex encapsulation layer. Gray serpentine channel and quasi-circular channel are the microfluidic channel with a line width of 300 μm. The serpentine design helps to collect tears so that the tears can completely wet the MNS; the yellow area and the pink area are Pt wires and Ag/AgCl electrodes with the line width of 300 μm and the arc length of ~1.2 mm; the blue area is the MNS electrode with the line width of 300 μm and the interval of 300 μm. **(d)** Transfer all of the above to the contact lens mold for packaging.

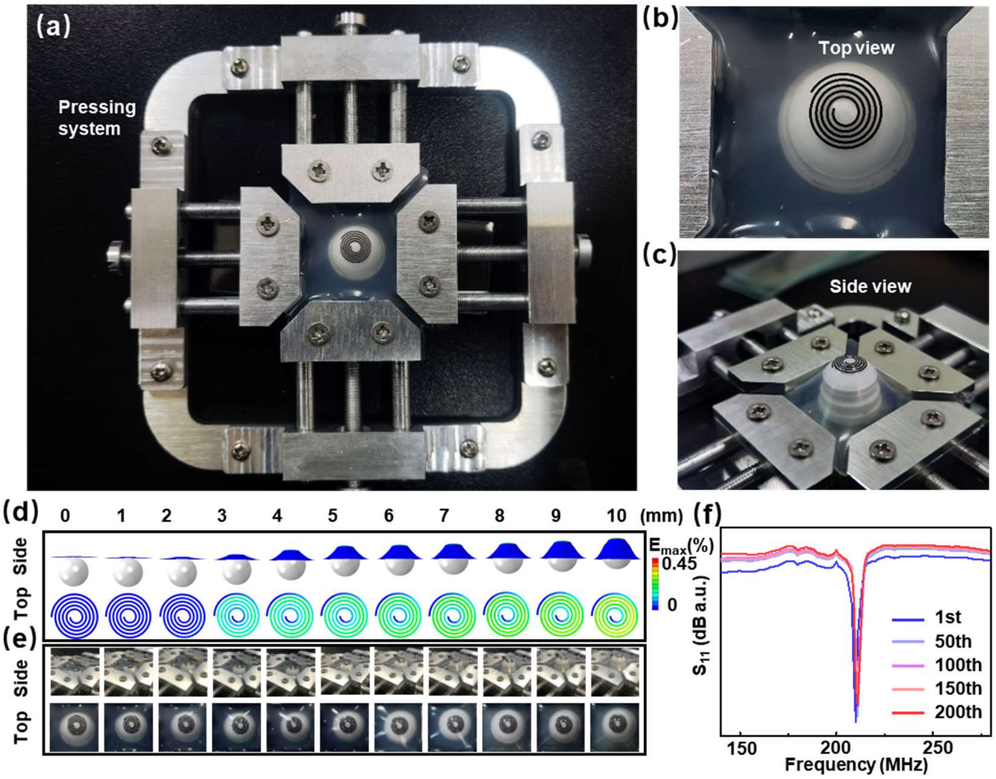


**Figure S2. Mechanical robustness of MCL. (a)** Optical image of the overall pressing system. **(b)** Top view and **(c)** Side view of the MCL pressed with a spherical plastic ball with a radius of 4 mm. **(d)** FEA and experimental results of the MCL under a series of pressing heights. **(e)** Corresponding height experimental results of the MCL. **(f)** Reflection coefficients for different deformation times.

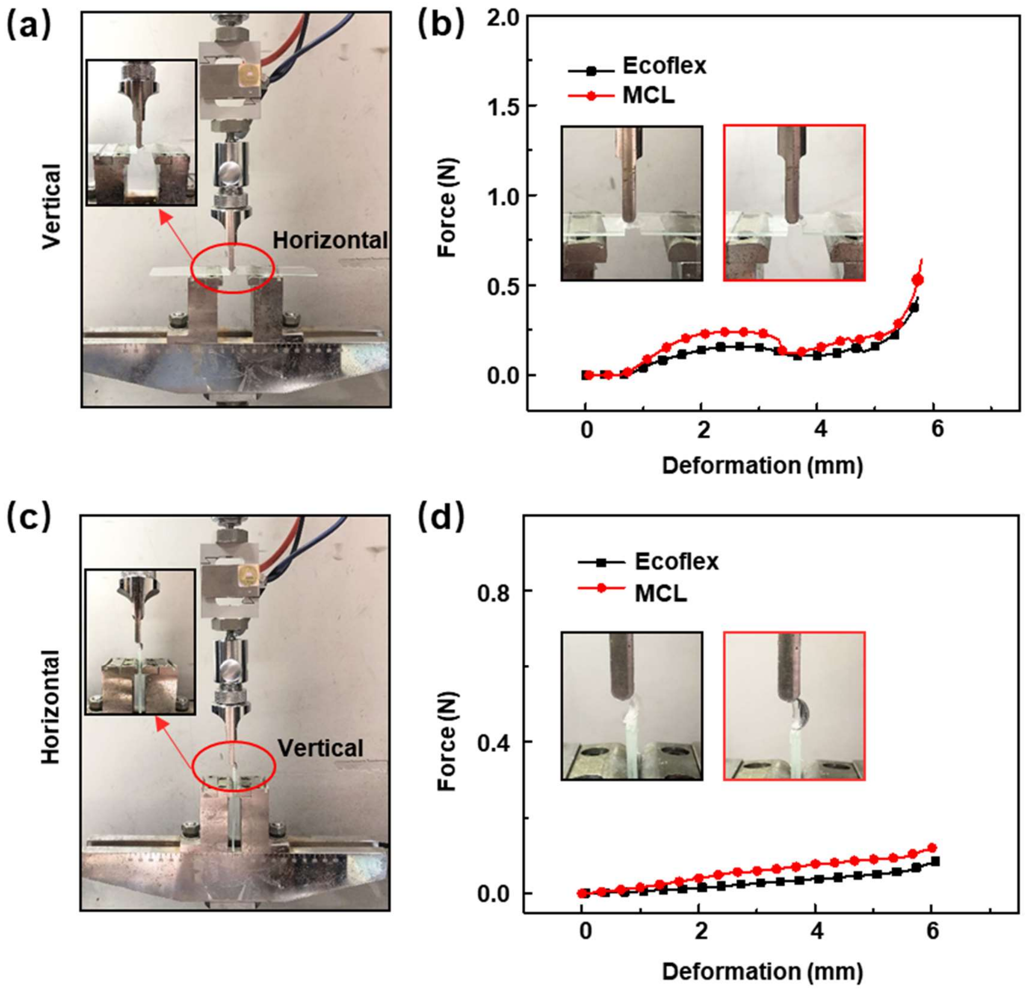


**Figure S3. Horizontal and vertical mechanical property of the MCL and BCL. (a)** Optical image of a universal testing machine for three-point bending test and the MCL is fixed horizontally on the support platform (red circle). **(b)** Bending stress versus deformation curves for MCL and BCL in the vertical direction. **(c)** Optical image of MCL fixed vertically on the support platform (red circle). **(d)** Bending stress versus deformation curves for MCL and BCL in the horizontal direction.

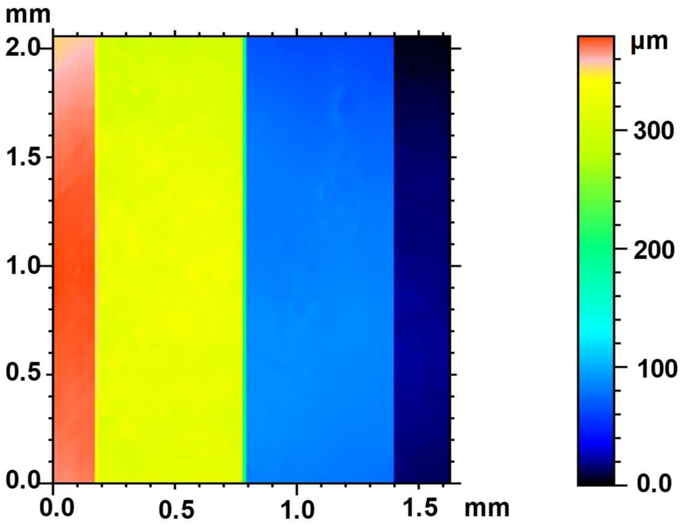


**Figure S4.** 3D microscope scanned image of MNS.

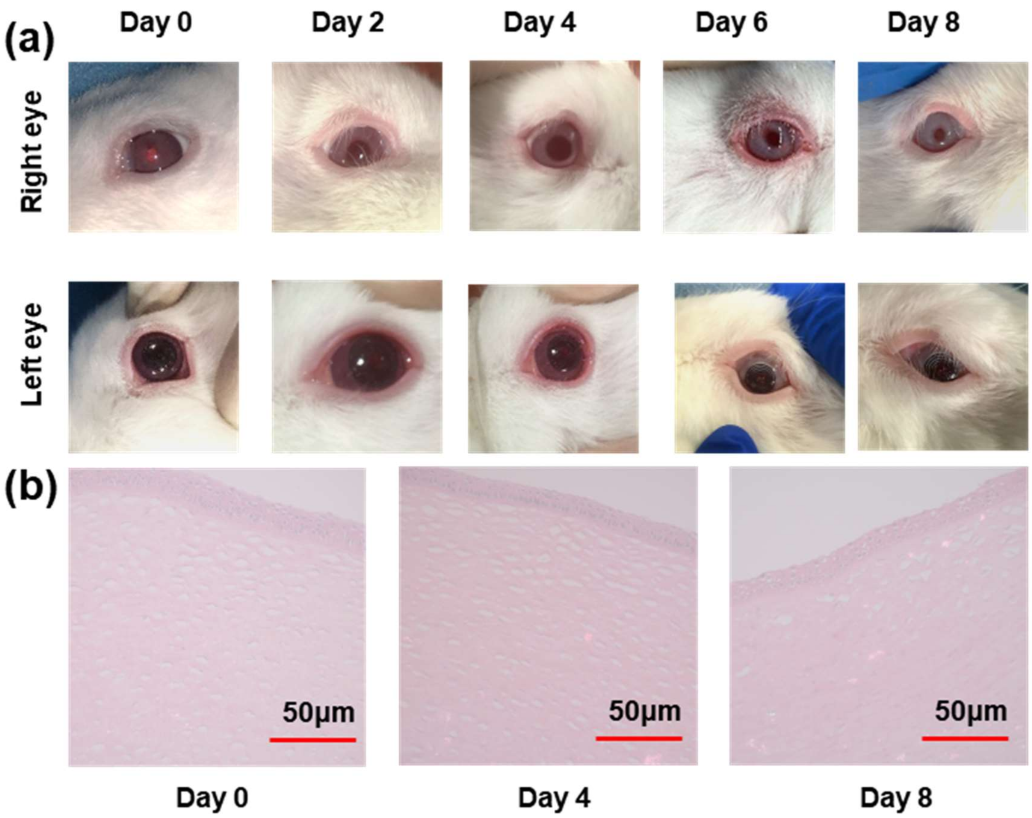


**Figure S5.** ***In vivo*** **biosafety of the MCL. (a)** Optical images of rabbit eyes for 8 days. The right eye is the control eye (without wearing MCL), the left eye is the experimental eye (continuously wearing MCL). **(b)** H&E stains of rabbit cornea with different wearing time points (left: 0 day, middle: 4 days, Right: 8 days).

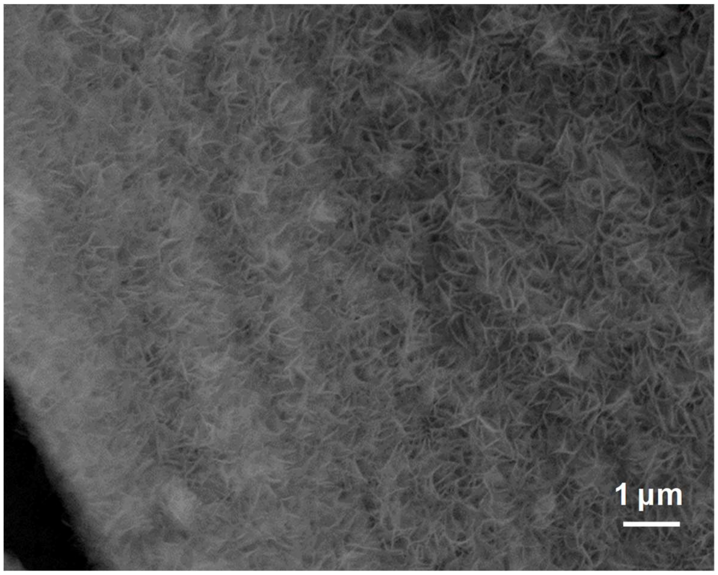


**Figure S6.** SEM image of the as-prepared MNS.

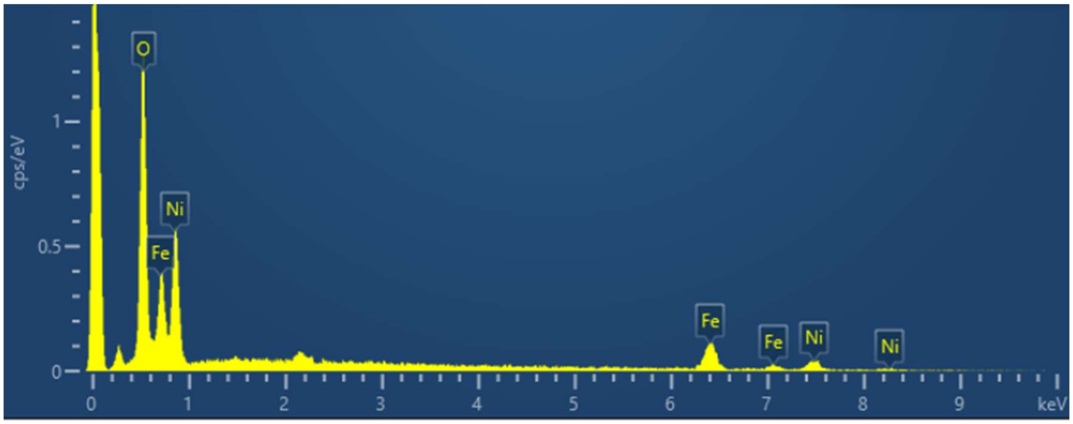


**Figure S7.** EDX spectrum of the as-prepared MNS.

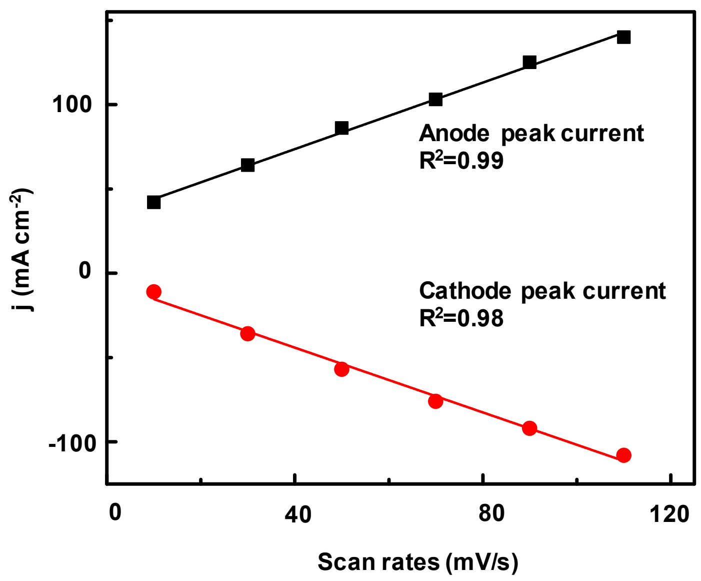


**Figure S8.** Linear relationship between the peak current (black line: anode; red line: cathode) of the electrode and different scan rates.

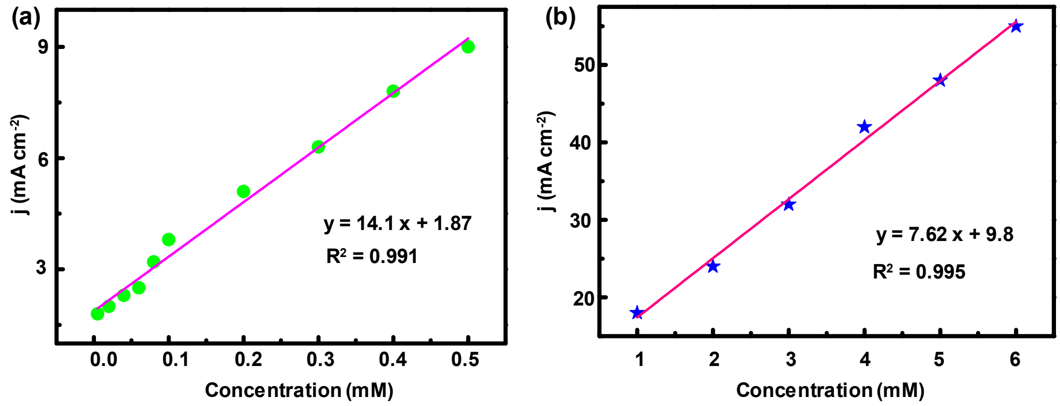


**Figure S9.** Linear relation of the current response with the concentration of glucose within the range of (a) 0.005-0.5mM glucose and (b) 1.0-6.0mM.

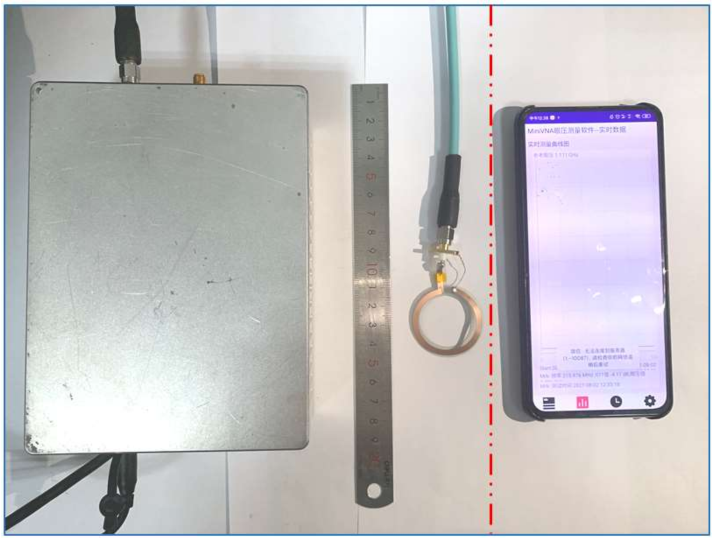


**Figure S10.** Images of the IOP test system. Wireless transmission includes portable network analyzer (left) and smart phone (right).

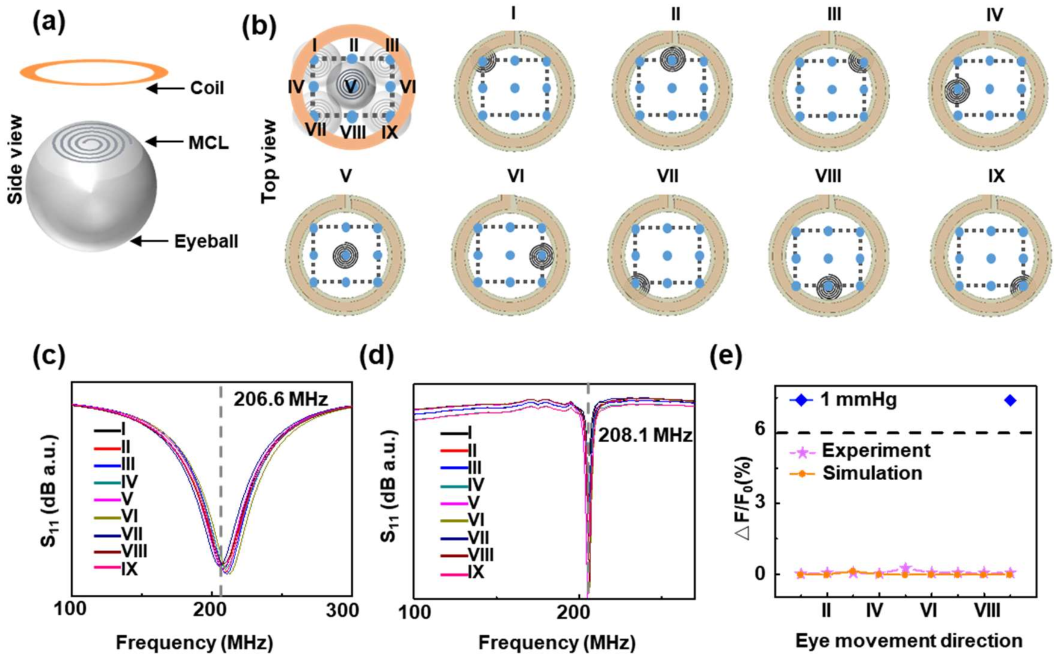


**Figure S11. Impacts of different locations on the performance of the IOP sensor.** **(a)** Schematic diagram of eyeball movement (side view). **(b)** Electromagnetic simulation images of the eyeball at nine locations (top view). **(c)** Electromagnetic simulation results of the MCL at different locations. **(d)** Experimental results of the MCL at different locations. **(e)** Comparison of relative deviations of eye movements in nine locations and elevated intraocular pressure of 1 mm Hg.

**Table S1.** Comparison of sensing performances of the as-prepared MNS with other Ni or Fe based nonenzymatic electrochemical glucose sensors.

| Catalysts | Linear range (mM) | Detection limit (μM) | Ref. |
|---|---|---|---|
| MNS | 0.005-0.5<br>1.0-6.0 | 0.43 | This work |
| $NiCo_2O_4$ hollow nanorods | 0.003-1 | 0.16 | 1 |
| FeBDC-derived $Fe_3O_4$ | 0-9 | 15.7 | 2 |
| $NiO/Fe_2O_3$ | 0.05-2.867 | 3.9 | 3 |
| NiO hollow nanosphere | 1.5-7.0 | 47 | 4 |
| $NiO/CeO_2$ nanoflake | 0.001-2.9 | 1.0 | 5 |
| NiO doped ZnO nanorods | 0.5-8 | 2.5 | 6 |
| $\gamma$-$Fe_2O_3$ nanoparticles | 0-1.5 | 20 | 7 |
| $Fe_3O_4$ nanoparticles | 0.006-2.2 | 6 | 8 |
| g-$C_3N_4/Fe_2O_3$-Cu | 0.006-2.0 | 0.3 | 9 |
| g-$C_3N_4/\alpha$-$Fe_2O_3$ | 0.002-2.4 | 0.4 | 10 |
| $\alpha$-$Fe_2O_3$/NF | 0.005-0.2 | 0.87 | 11 |
| $Fe_2O_3$-ZNRs | 0.05-18 | 12 | 12 |
| $Fe_2O_3$-NPs/$P_4$VP-co-PAN | 0.0025-0.58 | 0.58 | 13 |
| Hierarchical NiO | 0.018-1.2 | 6.15 | 14 |

**Table S2. Feature comparison between MCL and other wearable electronics for eye movement tracking.**

| Sensing materials | Manifestation | Monitoring principle | Ref. |
|---|---|---|---|
| MNS Coil | contact lenses | magnetic response | This work |
| hydrogel electrode (tAgTrode) | portable eye mask | EOG | 15 |
| AgNPs | VR device | EOG | 16 |
| Magnet | contact lenses | magnetoresistance effect | 17 |

**Table S3.** Comparison between this work and the previous IOP sensors.

| Sensing materials | IOP range (mmHg) | Sensitivity | Ref. |
|---|---|---|---|
| MNS Coil | 6-60 | 0.17 MHz $mmHg^{-1}$ | This work |
| Pt | 17-29 | 8.37 μV $mmHg^{-1}$ | 18 |
| Pt | 19-30 | 113μV $mmHg^{-1}$ | 19 |
| Self-assembled graphene | 16-32 | 3.166 mV $mmHg^{-1}$ | 20 |
| Cu | 5-40 | 23 kHz $mmHg^{-1}$ | 21 |
| CVD graphene | 10-21 | 150 μV $mmHg^{-1}$ | 22 |
| BL film | 0-22 | 1.5 Ω $mmHg^{-1}$ | 23 |
| Graphene/AgNWs | 0-50 | 2.64 MHz $mmHg^{-1}$ | 24 |
| Cu | 5-45 | 8 kHz $mmHg^{-1}$ | 25 |
| GWFs | 0-15 | 6.8% $mmHg^{-1}$ | 26 |
| PDMS | 0-30 | 50-137 μm $mmHg^{-1}$ | 27 |
| Ti/Pt | 9-30 | 20 μV $mmHg^{-1}$ | 28 |

## References


1. Yang J, Cho M, Lee Y. Synthesis of Hierarchical $NiCo_2O_4$ Hollow Nanorods via Sacrificial-Template Accelerate Hydrolysis for Electrochemical Glucose Oxidation. Biosens. and Bioelectron. 2016; 75:15-22.
2. Abrori S, Septiani N, Nugraha, Anshori I, Suyatman, Suendo V, Yuliarto B. Metal-Organic-Framework FeBDC-Derived $Fe_3O_4$ for Non-Enzymatic Electrochemical Detection of Glucose. Sensors. 2020; 20(17): 4891-4907.
3. Luo L, Cui J, Wang Y, WangY, Zheng H, Qin Y, Shu X, Yu D, Zhang Y, Wu Y. Synthesis of NiO/$Fe_2O_3$ Nanocomposites as Substrate for The Construction of Electrochemical Biosensors. J. Solid State Electr. 2018; 22(6):1763-1770.
4. Li C, Liu Y, Li L, Du Z, Xu S, Zhang M, Yin X, Wang T. A Novel Amperometric Biosensor Based on NiO Hollow Nanospheres for Biosensing Glucose. Talanta 2008; 77(1): 455-459.
5. Cui J, Luo J, Peng B, Zhang X, Zhang Y, Wang Y, Qin Y, Zheng H, Shu X, Wu Y. Synthesis of Porous NiO/$CeO_2$ Hybrid Nanoflake Arrays as A Platform for Electrochemical Biosensing. Nanoscale 2016; 8(2): 770-774.
6. Chu X, Zhu X, Dong Y, Chen T, Ye M, Sun W. An Amperometric Glucose Biosensor Based on The Immobilization of Glucose Oxidase on the Platinum Electrode Modified with NiO Doped ZnO Nanorods. Electroanal. Chem. 2012; 676: 20-26.
7. Baratella D, Magro M, Sinigaglia G, Zboril R, Salviulo G, Vianello F. A Glucose Biosensor Based On Surface Active Maghemite Nanoparticles. Biosens. and Bioelectron. 2013; 45: 13-18.
8. Yang L, Ren X, Tang F, Zhang L. A Practical Glucose Biosensor Based on $Fe_3O_4$ Nanoparticles and Chitosan/nafion Composite Film. Biosens. and Bioelectron. 2009; 25(4): 889-895.
9. Liu L, Wang M, Wang C. In-situ Synthesis of Graphitic Carbon Nitride/Iron Oxide−Copper Composites and Their Application in The Electrochemical Detection of Glucose. Electrochim. Acta 2018; 265: 275-283.
10. Liu L, Wang J, Wang C, Wang G. Facile Synthesis of Graphitic Carbon Nitride/Nanostructured α-$Fe_2O_3$ Composites and Their Excellent Electrochemical Performance for Supercapacitor and Enzyme-Free Glucose Detection Applications. Appl. Surf. Sci. 2016; 390: 303-310.
11. Liu Y, Zhao W, Li X, Liu J, Han Y, Wu J, Zhang X, Xu Y. Hierarchical α-$Fe_2O_3$

Microcubes Supported on Ni Foam as Non-Enzymatic Glucose Sensor. Appl. Surf. Sci. 2020; 512: 145710.

12. Ahmad R, Ahn M, Hahn Y. Fabrication of A Non-Enzymatic Glucose Sensor Field-Effect Transistor Based on Vertically-Oriented ZnO Nanorods Modified with $Fe_2O_3$. Electrochem. Commun. 2017; 77: 107-111.
13. Chen Y, Zhang H, Xue H, Hu X, Wang G, Wang C. Construction of A Non-Enzymatic Glucose Sensor Based on Copolymer P4VP-co-PAN and $Fe_2O_3$ Nanoparticles. Mat. Sci. Eng. C. 2014; 35: 420-425.
14. Wang L, Xie Y, Wei C, Lu X, Li X, Song Y. Hierarchical NiO Superstructures/Foam Ni Electrode Derived from Ni Metal-Organic Framework Flakes on Foam Ni for Glucose Sensing. Electrochim. Acta. 2015; 174: 846-852.
15. Homayounfar S, Rostaminia S, Kiaghadi A, Chen X, Alexander E, Ganesan D, Andrew T. Multimodal Smart Eyewear for Longitudinal Eye Movement Tracking. Matter. 2020; 3(4):1275-1293.
16. Mishra S, Kim Y, Intarasirisawat J, Kwon Y, Lee Y, Mahmood M, Lim H, Herbert R, Yu K, Ang C, Yeo W. Soft, Wireless Periocular Wearable Electronics for Real-time Detection of Eye Vergence in A Virtual Reality toward Mobile Eye Therapies. Sci. Adv. 2020; 6(11): eaay1729.
17. Tanwear A, Liang X, Liu Y, Vuckovic A, Ghannam R, Böhnert T, Paz E, Freitas P, Ferreira R, Heidari H. Spintronic Sensors Based on Magnetic Tunnel Junctions for Wireless Eye Movement Gesture Control. IEEE Transactions on Biomedical Circuits and Systems. 2020; 14(6): 1299-1310.
18. Leonardi M, Leuenberger P, Bertrand D, Bertsch A, Renaud P. First Steps toward Noninvasive Intraocular Pressure Monitoring with a Sensing Contact Lens. Investigative Ophthalmology & Visual Science. 2004; 45(9): 3113-3117.
19. Leonardi M, Pitchon E M, Bertsch A, Renaud P, Mermoud A. Wireless Contact Lens Sensor for Intraocular Pressure Monitoring: Assessment on Enucleated Pig Eyes. Acta Ophthalmologica. 2010; 87(4): 433-437.
20. Liu Z, Wang G, Ye C, Sun H, Pei W, Wei C, Dai W, Dou Z, Sun Q, Lin C, Wang Y, Chen H, Shen G. An ultrasensitive contact lens sensor based on self-assembly graphene for continuous intraocular pressure monitoring. Adv. Funct. Mater. 2021; 31:2010991.
21. Chen G, Chan I, Lam D. Capacitive Contact Lens Sensor for Continuous Non-

invasive Intraocular Pressure Monitoring. Sensor. Actuat. A. 2013; 203:112-118.
22. Xu J, Cui T, Hirtz T, Qiao Y, Li X, Zhong F, Han X, Yang Y, Zhang S, Ren T. Highly Transparent and Sensitive Graphene Sensors for Continuous and Non-invasive Intraocular Pressure Monitoring. ACS Appl. Mater. Interfaces. 2020; 12(16): 18375–18384
23. Laukhin V, Sánchez I, Moya A, Laukhina E, Martin R, Ussa F, Rovira C, Guimera A, Villa R, Aguiló J, Pastor J, Veciana J. Non-invasive Intraocular Pressure Monitoring with A Contact Lens Engineered with A Nanostructured Polymeric Sensing Film. Sensor. Actuat. A. 2011; 170(1-2): 36-43.
24. Kim J, Kim M, Lee M, Kim K, Ji S, Kim Y, Park J, Na K, Bae K-H, Kyun Kim H, Bien F, Young Lee C, Park J-U. Wearable Smart Sensor Systems Integrated on Soft Contact Lenses for Wireless Ocular Diagnostics. Nat. Commun. 2017; 8: 14997.
25. Chen G-Z, Chan I-S, Leung L, Lam D. Soft Wearable Contact Lens Sensor for Continuous Intraocular Pressure Monitoring. Med. Eng. Phys. 2014; 36(9): 1134-1139.
26. Zhang Y, Chen Y, Man T, Huang D, Li X, Zhu H, Li Z. High Resolution Non-Invasive Intraocular Pressure Monitoring by Use of Graphene Woven Fabrics on Contact Lens. Microsyst. Nanoeng. 2019; 5:39.
27. Araci I, Su B, Quake S, Mandel Y. An Implantable Microfluidic Device for Self-Monitoring of Intraocular Pressure. Nat. Med. 2014; 20: 1074-1078.
28. Yu P, Li Y, Wang X, Qi C, Ren T. A Contact Lens Promising for Non-invasive Continuous Intraocular Pressure Monitoring. RSC Adv. 2019; 9(9):5076-5082.
